# Classical Information and Collapse in Wigner’s Friend Setups

**DOI:** 10.3390/e25101420

**Published:** 2023-10-06

**Authors:** Veronika Baumann

**Affiliations:** 1 Atominstitut, Technische Universität Wien, 1020 Vienna, Austria; veronika.baumann@oeaw.ac.at; 2 Institute for Quantum Optics and Quantum Information (IQOQI), Boltzmanngasse 3, 1090 Vienna, Austria

**Keywords:** Wigner’s friend, classical information, quantum foundations

## Abstract

The famous Wigner’s friend experiment considers an observer—the friend—and a superobserver—Wigner—who treats the friend as a quantum system and her interaction with other quantum systems as unitary dynamics. This is at odds with the friend describing this interaction via collapse dynamics, if she interacts with the quantum system in a way that she would consider a measurement. These different descriptions constitute the Wigner’s friend paradox. Extended Wigner’s friend experiments combine the original thought experiment with non-locality setups. This allows for deriving local friendliness inequalities, similar to Bell’s theorem, which can be violated for certain extended Wigner’s friend scenarios. A Wigner’s friend paradox and the violation of local friendliness inequalities require that no classical record exists, which reveals the result the friend observed during her measurement. Otherwise, Wigner agrees with his friend’s description and no local friendliness inequality can be violated. In this article, I introduce classical communication between Wigner and his friend and discuss its effects on the simple as well as extended Wigner’s friend experiments. By controlling the properties of a (quasi) classical communication channel between Wigner and the friend, one can regulate how much outcome information about the friend’s measurement is revealed. This gives a smooth transition between the paradoxical description and the possibility of violating local friendliness inequalities, on the one hand, and the effectively collapsed case, on the other hand.

## 1. Introduction

The Wigner’s friend thought experiment was originally proposed in [1] to reason about the applicability of the two dynamics featured by quantum mechanics. On the one hand, sufficiently isolated quantum systems evolve unitarily. On the other hand, quantum systems upon measurement undergo a seemingly instantaneous collapse to the eigenstate corresponding to the observed outcome. These two different dynamics and the lack of a clear prescription of when to use one or the other is one of the main aspects of the quantum measurement problem [2,3,4].

**Figure 1:**
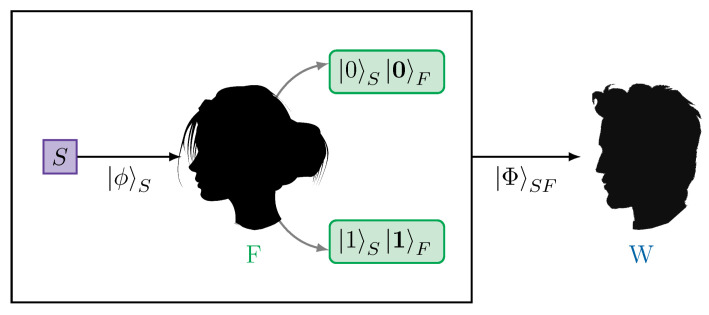
Simple Wigner’s friend experiment: The source emits a quantum state ${|\phi \rangle }_{S}$, which is measured by the friend in the computational basis {${|0\rangle }_{S},{|1\rangle }_{S}$}. The result observed by the friend is stored in some memory register ${|\cdot \rangle }_{F}$ and she ascribes the respective product ${|i\rangle }_{S}{|i\rangle }_{F}$, with i∈{0,1}, to herself and the system she measured. Wigner performs a measurement on both the system and the friend’s memory register, which, according to his unitary description, is in state ${|\Phi \rangle }_{SF}$ that is, in general, a superposition of ${|0\rangle }_{S}{|0\rangle }_{F}$ and ${|1\rangle }_{S}{|1\rangle }_{F}$. The different state assignments to the joint system S+F will lead to different probability assignments for Wigner’s measurement.

The original thought experiment features an observer—called Wigner’s friend—who measures a quantum system, as well as a so-called superobserver—Wigner—who performs a joint measurement on the quantum system S and the friend F. Provided that the joint system S + F is sufficiently isolated, Wigner describes the friend’s interaction with the system via unitary dynamics. To Wigner’s friend, however, this interaction constitutes a measurement and she uses the collapse postulate after observing an outcome. These disagreeing descriptions of one and the same situation are called the Wigner’s friend paradox, see Figure 1. Let the source emit a qubit in the state ${|\phi \rangle }_{S}=\alpha {|0\rangle }_{S}+\beta {|1\rangle }_{S}$, which is measured by the friend in the computational basis {|0〉,|1〉}. The result she observes, f∈{0,1}, is stored in her memory register ${|\cdot \rangle }_{F}$, which is supposed to correspond to the friend having a perception of the outcome *f*. Wigner then performs a measurement on the qubit and the friend’s memory given by the states ${|1\rangle }_{SF}=a{|0,0\rangle }_{SF}+b{|1,1\rangle }_{SF}$ and ${|2\rangle }_{SF}=b^{*}{|0,0\rangle }_{SF}-a^{*}{|1,1\rangle }_{SF}$ and their orthogonal complement. Due to their different descriptions of the friend’s measurement, Wigner and his friend will assign different probabilities to the outcomes of Wigner’s measurement. According to the friend, after her measurement, the qubit and her memory are either in state ${|0,0\rangle }_{SF}$, which happens with probability $p\left(0\right)={\left|\alpha \right|}^{2}$, or in state ${|1,1\rangle }_{SF}$, which happens with probability $p\left(1\right)={\left|\beta \right|}^{2}$. Hence, the friend will assign the following probability to Wigner’s results

(1) $$p^{F}\left(w\right)=p^{clps}\left(w\right)={\left|\alpha \right|}^{2}{\left|\langle w|0,0\rangle \right|}^{2}+{\left|\beta \right|}^{2}{\left|\langle w|1,1\rangle \right|}^{2},$$

where |w〉 is either ${|1\rangle }_{SF}$ or ${|2\rangle }_{SF}$. Wigner, however, assigns the state ${|\Phi \rangle }_{SF}=\alpha {|0,0\rangle }_{SF}+\beta {|0,0\rangle }_{SF}$ to the qubit and his friend’s memory and, hence, probabilities

(2) $$p^{W}\left(w\right)=p^{uni}\left(w\right)={\left|{\langle w|\Phi \rangle }_{SF}\right|}^{2}.$$

More concretely, we obtain the following predictions for Wigner’s measurement according to Wigner and his friend in the simple Wigner’s friend experiment

(3) pW(w):12(αa*+βb*)2(βa−αb)2      pF(w):12|α|2|a|2+|β|2|b|2|β|2|a|2+|α|2|b|2.

When the experiment is performed, at most one of these probability assignments can agree with the observed frequencies. If the friend’s predictions turn out to be correct, this means that unitary dynamics do not describe observations. If, on the other hand, Wigner’s predictions agree with empirical observation, this means that the state update rule does not apply for all types of measurements. Wigner originally argued in favor of the friend’s description (and probability assignment) claiming that at the level of an observer, at the latest, unitary quantum theory must break down. Since then, however, the idea of observers in superposition has become accepted [5], which begs the question of whether the disagreement between Wigner and his friend can be experimentally verified. As already discussed in [6,7,8], the unitary description of the friend’s measurement is incompatible with the existence of a record revealing which result the friend observed before Wigner performs his measurement. Such a record destroys any coherences Wigner could reveal in his measurement. However, there are persistent records that do not contain any outcome information of the friend’s measurement and can, therefore, be present without destroying the coherence of an observer in superposition [5,8]. In special cases, the disagreement between Wigner and his friend becomes manifested in terms of contradicting records that both Wigner and his friend can access after the thought experiment has been performed. Note that, also in these cases, there is no persistent record of which result the friend observed at her measurement since Wigner’s measurement will, in general, alter the friend’s perception of her measurement result, i.e., the result stored in the internal memory register [9].

**Figure 2:**
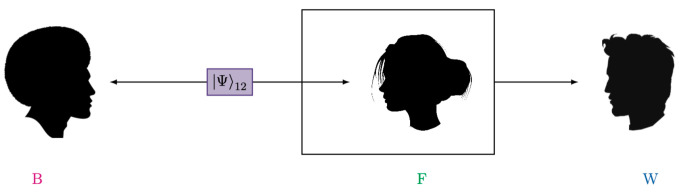
Simplest extended Wigner’s friend experiment: A bipartite state ${|\Psi \rangle }_{12}$ is emitted by the source. One subsystem is measured by the friend—F—in a simple Wigner’s friend setup. Together with the subsystem she interacted with, the friend is then measured by Wigner—W. The other subsystem is measured by an additional observer, Bob—B.

The newest versions of the thought experiment combine multiple Wigner’s friend scenarios with various non-locality proofs [10,11,12,13,14,15,16,17]. These extended Wigner’s friend experiments led to striking no-go theorems for seemingly natural assumptions about quantum theory and observation. Some of these no-go theorems, for example [16], rely on conflicting probability assignments of observers and superobservers similar to the simple Wigner’s friend experiment above. More concretely, combining the predictions of multiple (entangled) observers and superobservers according to a set of assumptions gives a contradiction. This led to closer investigations of how agents in Wigner’s-friend-like situations can make predictions and consistently reason about each other [18,19]. Other extended Wigner’s friend scenarios concern the joint probabilities of the results of observers and superobservers, similar to Bell’s theorem. More specifically, a set of assumptions called local friendliness—namely, that the superobservers’ and observers’ results are both “objective facts”, locality, freedom of choice, and universality of quantum theory, meaning observers can exist in superpositions of different observation states—cannot all hold in extended Wigner’s friend setups. Other than the disagreement between observers and superobservers about the probabilities of individual measurement outcomes, the violation of local friendliness inequalities asserts that any joint probability distribution one can assign to the respective outcomes cannot be of the form implied by the assumptions. The local friendliness no-go theorem can, therefore, not be resolved by giving a prescription of how observers should make predictions in Wigner’s friend setups. Like in the case of Bell’s theorem, in the face of violations of the respective inequalities, one is left with the decision of which of the assumptions one wants to reject. In the simplest extended Wigner’s friend scenario, as depicted in Figure 2, a bipartite entangled state is shared between a Wigner’s friend setup and one additional observer—Bob. The violation of a CHSH-like-inequality between Wigner and Bob asserts that the local friendliness assumptions cannot hold simultaneously.

Consider the setup in Figure 2, where Wigner randomly chooses between the measurement that reveals which result his friend observed in her measurement $W_{z}={\left|0,0\rangle \langle 0,0\right|}_{2F}-{\left|1,1\rangle \langle 1,1\right|}_{2F}$ and one projecting on the states ${|\Phi^{\pm }\rangle }_{2F}=1/\sqrt{2}\left({|0,0\rangle }_{2F}\pm {|1,1\rangle }_{2F}\right)$, i.e., $W_{x}=|0,0\rangle \langle {1,1|}_{2F}+|1,1\rangle \langle {0,0|}_{2F}$. Bob, on the other hand, performs measurements $B_{z}=1\sqrt{2}(|0\rangle \langle 0|+|0\rangle \langle 1|+|1\rangle \langle 0|-|1\rangle \langle 1|)$ and $B_{x}=1\sqrt{2}(|0\rangle \langle 0|-|0\rangle \langle 1|-|1\rangle \langle 0|-|1\rangle \langle 1|)$ on qubit 1. One local friendliness inequality for this scenario is given by the following CHSH-expression

(4) $$\langle B_{z}⊗W_{z}\rangle +\langle B_{x}⊗W_{z}\rangle -\langle B_{z}⊗W_{x}\rangle +\langle B_{x}⊗W_{x}\rangle \leq 2.$$

Other inequalities can be obtained by making an alternative choice for which expectation value is subtracted on the left-hand side. If the source emits the singlet state ${|\psi^{-}\rangle }_{12}=1/\sqrt{2}\left({|0,0\rangle }_{12}-{|1,1\rangle }_{12}\right)$ and the friend measures qubit 2 in the computational basis, we obtain the overall state

(5) $$|\Psi \rangle =\frac{1}{\sqrt{2}}\left({|0\rangle }_{1}{|0,0\rangle }_{2F}-{|1\rangle }_{1}{|1,1\rangle }_{2F}\right)$$

on which Wigner and Bob perform their measurements. This gives the following violation of the inequality in Equation (4)

(6) $$\langle B_{z}⊗W_{z}\rangle +\langle B_{x}⊗W_{z}\rangle -\langle B_{z}⊗W_{x}\rangle +\langle B_{x}⊗W_{x}\rangle =4\cdot \frac{1}{\sqrt{2}}=2\sqrt{2}>2.$$

The violations of local friendliness inequalities have been confirmed experimentally in the proof of principle experiments [14,20] where an additional qubit played the role of Wigner’s friend. Such experiments arguably do not constitute genuine Wigner’s friend experiments, since the interaction between two qubits does not satisfy many basic characteristics of an observation [21]. In response to that, an extended Wigner’s friend experiment involving a human-level AI on a quantum computer playing the role of the friend has been proposed [22]. Such a friend would satisfy most qualitative features of an observer while operating fully unitarily by construction. In this version of a Wigner’s friend experiment, the AI friend generates a message after interacting with the system in each round. The superobserver then sends back this system and reverses the message generation. In this article, we examine the possibility of retaining such messages and the effect that has on local friendliness inequalities.

The rest of this article is structured as follows. In the main Section 2, I consider communication between Wigner and his friend first by incorporating record systems that are not subject to Wigner’s measurement in Section 2.1. Second, I introduce a (quasi) classical communication channel into the simple Wigner’s friend experiment in Section 2.2. This allows me to recover probabilities in agreement with collapse dynamics as well as with unitary dynamics and anything in between depending on the properties of the communication channel. In Section 2.3, I then consider the records as well as the communication channel in an extended Wigner’s friend setup and discuss their implications on the violation of the local friendliness inequality presented above. Finally, the conclusions are summarized in Section 3.

## 2. Classical Information and Collapse

As discussed in [5,7,23], unitary dynamics is incompatible with simultaneously preserving a classical record of a measurement result. For Wigner’s friend experiments, this means that there cannot be a record revealing the friend’s observed outcome. Conversely, certain notions of classicality have been invoked to define an observer and, based on that, reject the unitary description of observers [24,25]. Here, we denote any carrier of classical information as a classical record. In particular, this allows us to consider quantum systems with a fixed basis as a classical record. In general, such records or messages that are not subject to Wigner’s measurement influence the probabilities for Wigner’s results according to a unitary description of the setup.

### 2.1. Effective Collapse

If the friend produces a classical record revealing her observed result, then Wigner’s description of the setup gives rise to the probabilities induced by collapse dynamics. Consider a record Hilbert space $H_{R}$ with a fixed basis {$|r_{i}\rangle$}, which encodes the (quasi) classical messages Wigner receives from his friend. For example, in the simplest Wigner’s friend experiment in Figure 1, these messages could correspond to $|r_{0}\rangle =|‘‘I\;\mathrm{saw}\;0.’’\rangle$ and $|r_{1}\rangle =|‘‘I\;\mathrm{saw}\;1.’’\rangle$. A unitary description of the setup, then, leads to the following overall state

(7) $$|\Psi^{r}\rangle =\alpha {|0,0\rangle }_{SF}{|r_{0}\rangle }_{R}+\beta {|1,1\rangle }_{SF}{|r_{1}\rangle }_{R},$$

upon which Wigner performs his measurement. The probabilities for Wigner’s measurement result are then given by

(8) $$p^{W}\left(w\right)=\left({\left|w\rangle \langle w\right|}_{SF}|\Psi^{r}\rangle \langle \Psi^{r}|\right),$$

which is equal to the ones assigned by the friend who uses collapse dynamics

(9) $$\begin{array}{c} p^{W}\left(w\right)=p^{F}\left(w\right): \end{array}\begin{array}{cc} 1 & 2\\ {\left|\alpha \right|}^{2}{\left|a\right|}^{2}+{\left|\beta \right|}^{2}{\left|b\right|}^{2} & {\left|\beta \right|}^{2}{\left|a\right|}^{2}+{\left|\alpha \right|}^{2}{\left|b\right|}^{2}. \end{array}$$

Note that, tracing out the record system can be understood as Wigner ignoring the record of the friend’s result. Yet, the existence of such a message alone, even if Wigner does not know what it reads, effectively collapses the state of the system and the friend. Such an effective collapse renders different ontological commitments about a quantum measurement empirically equivalent. Wigner could maintain that the friend’s measurement is in fact an entangling unitary, while the friend asserts that her measurement in fact collapses the state. If there exists a classical record of the friend’s result, this disagreement between Wigner and his friend no longer leads to disagreeing probability assignments and can, hence, not be decided empirically.

When taking into account which result the friend observed, we need to consider conditional probabilities. In the collapse description employed by the friend, this means either using the state |0,0〉 or |1,1〉 when calculating $p^{F}\left(w\right]f)={\left|\langle w|f,f\rangle \right]}^{2}$. In Wigner’s unitary description, we condition the record by evaluating probabilities

(10) $$\begin{array}{c} p^{W}\left(w,j\right)=\mathrm{Tr}\{{\left|w\rangle \langle w\right|}_{SF}⊗|r_{j}\rangle \langle r_{j}{|}_{R}|\Psi^{r}\rangle \langle \Psi^{r}|\}=p\left(j\right)p^{W}\left(w|j\right), \end{array}$$

where $p\left(j\right)=(1⊗|r_{j}\rangle \langle r_{j}{|}_{R}|\Psi^{r}\rangle \langle \Psi^{r}|)$ is the probability of message $r_{j}$ and we define p(w|j)=0 if p(j)=0. This gives

(11) pW(w|0)=pF(w]f=0):12|a|2|b|2      pW(w|1)=pF(w]f=1):12|b|2|a|2.

if the record reveals which result the friend observed. Conversely, if the record space is only one dimensional, for example, $|r^{\prime }\rangle =|‘‘I\;\mathrm{saw}\;a\;\mathrm{definite}\;\mathrm{outcome}’’\rangle$, the record factors out and

(12) $$|\Psi^{r^{\prime }}\rangle =\left(\alpha {|0,0\rangle }_{SF}+\beta {|1,1\rangle }_{SF}\right){|r^{\prime }\rangle }_{R},$$

which preserves the disagreement between Wigner and his friend. Conditioning on the record is obsolete in this case and the probabilities $p^{W}\left(w\right)=\left({\left|w\rangle \langle w\right|}_{SF}|\Psi^{r^{\prime }}\rangle \langle \Psi^{r^{\prime }}|\right)\neq p^{F}\left(w\right)$ are again those in Equation (3). This is due to the fact that such a one-dimensional record cannot reveal any outcome information of the friend’s two-outcome measurement.

### 2.2. Partial Collapse

**Figure 3:**
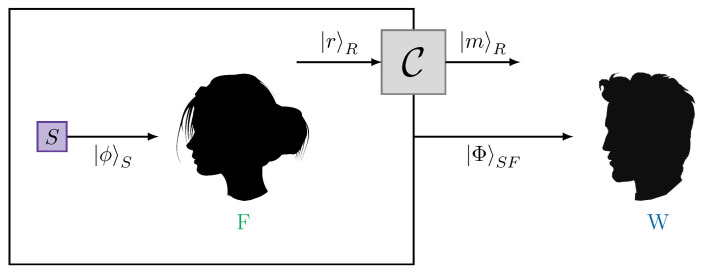
Communication channel C between Wigner and his friend. The friend can encode a message via a fixed basis {|r〉} of some record Hilbert space $H_{R}$. The channel C takes these record states as input and produces a classical message, which can, in principle, be encoded in another basis { |m〉} of the record Hilbert space. This freedom to choose different bases for the incoming and outgoing messages allows for controlling how much outcome information the friend can send to Wigner via this channel.

We now consider a more general scenario, where instead of directly exchanging messages, there is a (quasi) classical communication channel [26] between Wigner and his friend, see Figure 3. Such a channel measures the incoming state and prepares a corresponding outcome in some fixed basis {|n〉} of the record Hilbert space $H_{R}$

(13) $$C\left[\sigma \right]:=\sum_{m}\langle m|\sigma |m\rangle |m\rangle \langle m|.$$

In contrast to Section 2.1, the messages |m〉 sent to Wigner can now be encoded in a different basis of $H_{R}$ than the records |r〉 the friend produces. This allows for these messages to only partially reveal which outcome the friend observed. Another way of obtaining partial information about the system and the friend is by having Wigner perform weak measurements, which has been done in [27]. The state Wigner performs his measurement on is now

(14) $$\begin{array}{cc} \rho_{SFR}= & \left(1⊗C\right)|\Psi^{r}\rangle \langle \Psi^{r}|=\sum_{m}{\left|\alpha \right|}^{2}{\left|\langle m|r_{0}\rangle \right|}^{2}\rho_{SF}^{00}⊗{\left|m\rangle \langle m\right|}_{R}+{\left|\beta \right|}^{2}{\left|\langle m|r_{1}\rangle \right|}^{2}\rho_{SF}^{11}⊗{\left|m\rangle \langle m\right|}_{R}\\ \; & +\alpha \beta^{*}\langle m|r_{0}\rangle \langle r_{1}|m\rangle \rho_{SF}^{01}⊗{\left|m\rangle \langle m\right|}_{R}+\alpha^{*}\beta \langle m|r_{1}\rangle \langle r_{0}|m\rangle \rho_{SF}^{10}⊗{\left|m\rangle \langle m\right|}_{R}, \end{array}$$

where

$$\begin{array}{cc} \rho_{SF}^{00} & ={\left|a\right|}^{2}{\left|1\rangle \langle 1\right|}_{SF}+{\left|b\right|}^{2}{\left|2\rangle \langle 2\right|}_{SF}+a^{*}b^{*}12_{SF}+ab21_{SF},\\ \rho_{SF}^{11} & ={\left|b\right|}^{2}{\left|1\rangle \langle 1\right|}_{SF}+{\left|a\right|}^{2}{\left|2\rangle \langle 2\right|}_{SF}-a^{*}b^{*}12_{SF}-ab21_{SF},\\ \rho_{SF}^{01} & =a^{*}{b(|1\rangle \langle 1|}_{SF}-|2\rangle \langle 2{|}_{SF})-a^{*}a^{*}12_{SF}+bb21_{SF},\\ \rho_{SF}^{10} & =b^{*}{a(|1\rangle \langle 1|}_{SF}-|2\rangle \langle 2{|}_{SF})+b^{*}b^{*}12_{SF}-aa21_{SF}, \end{array}$$

with ${|1\rangle }_{SF}=a{|0,0\rangle }_{SF}+b{|1,1\rangle }_{SF}$, ${|2\rangle }_{SF}=b^{*}{|0,0\rangle }_{SF}-a^{*}{|1,1\rangle }_{SF}$ being the eigenstates corresponding to Wigner’s measurement results “1” and “2”, respectively. Similar to Equation (10) in Section 2.1, we consider the conditional probabilities of Wigner’s result *w* given message *n*, i.e., $p\left(n\right)p\left(w|n\right)={(|w\rangle \langle w|}_{SF}⊗|n\rangle \langle n{|}_{R}\rho_{SFR})$, obtaining the following joint probabilities

(15) $$\begin{array}{cc} p(w=1,n) & p(w=2,n)\\ {\left|\alpha \right|}^{2}{\left|a\right|}^{2}{\left|\langle n|r_{0}\rangle \right|}^{2}+{\left|\beta \right|}^{2}{\left|b\right|}^{2}{\left|\langle n|r_{1}\rangle \right|}^{2}\; & {\left|\beta \right|}^{2}{\left|a\right|}^{2}{\left|\langle n|r_{0}\rangle \right|}^{2}+{\left|\alpha \right|}^{2}{\left|b\right|}^{2}{\left|\langle n|r_{1}\rangle \right|}^{2}\\ +\alpha \beta^{*}a^{*}b\langle n|r_{0}\rangle \langle r_{1}|n\rangle +\alpha^{*}\beta ab^{*}\langle n|r_{1}\rangle \langle r_{0}|n\rangle \; & -\alpha \beta^{*}a^{*}b\langle n|r_{0}\rangle \langle r_{1}|n\rangle -\alpha^{*}\beta ab^{*}\langle n|r_{1}\rangle \langle r_{0}|n\rangle . \end{array}$$

The overlaps $\langle n|r_{i}\rangle$ indicate how much outcome information Wigner can obtain from the channel output message *n*. If the basis {|n〉} is the same as {$|r_{i}\rangle$}, i.e., $\langle n|r_{i}\rangle =\delta_{ni}$, the message perfectly reveals which result the friend observed and $p\left(n=0\right)={\left|\alpha \right|}^{2}$, $p\left(n=1\right)={\left|\beta \right|}^{2}$. In this case, we recover the collapse behavior in Equation (11), as discussed in Section 2.1. However, if the two bases are mutually unbiased, for example, $\langle 0|r_{i}\rangle =\langle 1|r_{0}\rangle =1/\sqrt{2}=-\langle 1|r_{1}\rangle$, the records reveal no outcome information about the friend’s measurement and p(n)=1/2 for both *n*. In this case, we obtain probabilities in accordance with a unitary description for each of the records

(16) p(w|0):12(αa*+βb*)2(βa−αb)2      p(w|1):12(αa*−βb*)2(βa+αb)2.

Note that the phase shift between the expressions for the two messages does not allow for simply adding them when wanting to preserve the resemblance to the unitary description without records. Evaluating p(w|0)+p(w|1) corresponds to tracing out the record system and, as already mentioned in Section 2.1, gives the collapse probabilities in Equation (9). In general, we can express the messages Wigner receives on the basis of the records generated by the friend as follows

(17) $$\begin{array}{cc} |0\rangle & =\cos\theta |r_{0}\rangle +e^{i\phi }\sin\theta |r_{1}\rangle \end{array}$$

(18) $$\begin{array}{cc} |1\rangle & =e^{-i\phi }\sin\theta |r_{0}\rangle -\cos\theta |r_{1}\rangle , \end{array}$$

where one can think of θ,ϕ as variable parameters of the communication channel C. Hence, the joint probabilities for Wigner’s measurement result and message *n* are given by

(19) $$\begin{array}{c} p(w,0): \end{array}\begin{array}{cc} 1 & 2\\ {\left|\alpha \right|}^{2}{\left|a\right|}^{2}\cos^{2}\left(\theta \right)+{\left|\beta \right|}^{2}{\left|b\right|}^{2}\sin^{2}\left(\theta \right)\; & {\left|\beta \right|}^{2}{\left|a\right|}^{2}\cos^{2}\left(\theta \right)+{\left|\alpha \right|}^{2}{\left|b\right|}^{2}\sin^{2}\left(\theta \right)\\ +\sin\left(2\theta \right)\left(\alpha \beta^{*}a^{*}be^{i\phi }+\alpha^{*}\beta ab^{*}e^{-i\phi }\right)\; & -\sin\left(2\theta \right)\left(\alpha \beta^{*}a^{*}be^{i\phi }+\alpha^{*}\beta ab^{*}e^{-i\phi }\right) \end{array}$$

and

(20) $$\begin{array}{c} p(w,1): \end{array}\begin{array}{cc} 1 & 2\\ {\left|\alpha \right|}^{2}{\left|a\right|}^{2}\sin^{2}\left(\theta \right)+{\left|\beta \right|}^{2}{\left|b\right|}^{2}\cos^{2}\left(\theta \right)\; & {\left|\beta \right|}^{2}{\left|a\right|}^{2}\sin^{2}\left(\theta \right)+{\left|\alpha \right|}^{2}{\left|b\right|}^{2}\cos^{2}\left(\theta \right)\\ -\sin\left(2\theta \right)\left(\alpha \beta^{*}a^{*}be^{i\phi }+\alpha^{*}\beta ab^{*}e^{-i\phi }\right)\; & +\sin\left(2\theta \right)\left(\alpha \beta^{*}a^{*}be^{i\phi }+\alpha^{*}\beta ab^{*}e^{-i\phi }\right). \end{array}$$

**Figure 4:**
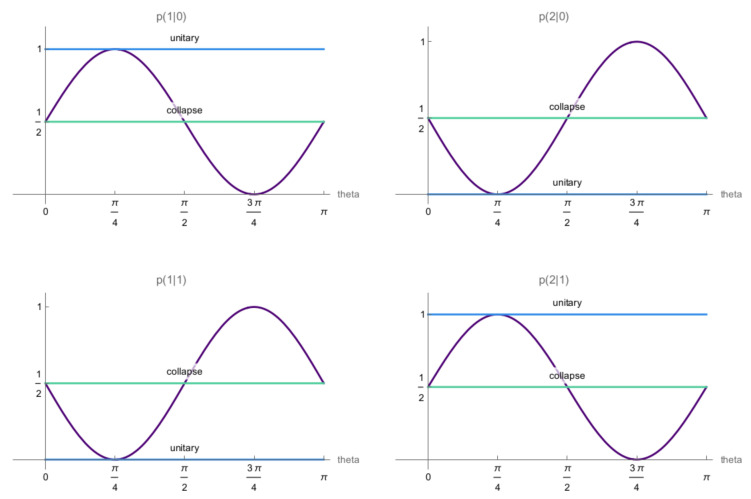
Partial collapse of communication between Wigner and his friend. If the source in the setup depicted in Figure 3 emits the state $|\phi \rangle =1/\sqrt{2}\left(|0\rangle +|1\rangle \right)$ and Wigner measures in the Bell-basis {$|\phi^{+}\rangle ,|\phi^{-}\rangle ,|\psi^{+}\rangle ,|\psi^{-}\rangle$}, he will obtain results “+” and “−” corresponding to states $|\phi^{+}\rangle =1/\sqrt{2}(|0,0\rangle +|1,1\rangle$ and $|\phi^{-}\rangle 1/\sqrt{2}(|0,0\rangle -|1,1\rangle$, respectively. For a communication channel C(θ,ϕ), and with ϕ=0, the probabilities for Wigner’s outcomes according to the unitary description of the setup depend on parameter θ, as shown above. These probabilities (violet) vary between those corresponding to the effectively collapsed state (green) and the effectively unitary case (blue).

Varying θ and ϕ then allows for smoothly changing between the effectively collapsed case in Equation (11) and the effectively unitary case in Equation (16), see Figure 4 for a simple example. Note that, for the partially collapsed scenarios there is still a paradoxical situation since the friend, when describing her measurement via collapse dynamics, will always assign the probabilities in Equation (11). Wigner’s probability assignments, namely those derived from Equations (19)–(20), will always differ from his friend’s unless the messages fully reveal which outcome the friend observed.

### 2.3. Local Friendliness Inequalities and Communication

Analogous to the simple Wigner’s friend experiment, the presence of classical records of the friend’s observed result effectively collapses the state Wigner performs his measurement on also in the extended Wigner’s friend setup depicted in Figure 2. This, in turn, prevents the violation of the local friendliness inequality presented in Section 1. More concretely, using state

(21) $$|\Psi^{r}\rangle =\frac{1}{\sqrt{2}}\left({|0\rangle }_{1}{|0,0\rangle }_{2F}{|r_{0}\rangle }_{R}-{|1\rangle }_{1}{|1,1\rangle }_{2F}{|r_{1}\rangle }_{R}\right)$$

instead of the one in Equation (5), leads to the following expression for the CHSH-like local friendliness inequality

(22) $$\langle B_{z}⊗W_{z}\rangle +\langle B_{x}⊗W_{z}\rangle -\langle B_{z}⊗W_{x}\rangle +\langle B_{x}⊗W_{x}\rangle =\frac{1}{\sqrt{2}}+\frac{1}{\sqrt{2}}+0+0=\sqrt{2}<2,$$

which means that none of the local friendliness assumptions need to be rejected. This is due to the fact that the presence of the records, revealing which result the friend observed, effectively collapses the state in Equation (21) to

(23) $${\mathrm{Tr}}_{R}(|\Psi^{r}\rangle \langle \Psi^{r}|)=\frac{1}{2}\left({\left|0\rangle \langle 0\right|}_{1}{\left|0,0\rangle \langle 0,0\right|}_{2F}+{\left|1\rangle \langle 1\right|}_{1}{\left|1,1\rangle \langle 1,1\right|}_{2F}\right),$$

which means that any expectation value containing Wigner’s $W_{x}$-measurement vanishes. Note that, this is also true when we condition on the friend’s observed result, meaning that we use either ${\left|0\rangle \langle 0\right|}_{1}{\left|0,0\rangle \langle 0,0\right|}_{2F}$ or ${\left|1\rangle \langle 1\right|}_{1}{\left|1,1\rangle \langle 1,1\right|}_{2F}$ as the effective state.

We now, again, consider a general communication channel C between the friend and Wigner, as depicted in Figure 3, also for this extended setup. Starting from the state in Equation (21), we now let the channel act on the register space and obtain the state

(24) $$\begin{array}{cc} \rho_{12FR}^{\prime } & =\left(1⊗C\right)\left(|\Psi^{r}\rangle \langle \Psi^{r}|\right)\\ \; & =\frac{1}{2}{\left(|0\rangle \langle 0\right|}_{1}⊗{\left|0,0\rangle \langle 0,0\right|}_{2F}⊗C(|r_{0}\rangle \langle r_{0}|)-|0\rangle \langle {1|}_{1}⊗|0,0\rangle \langle {1,1|}_{2F}C\left(|r_{0}\rangle \langle r_{1}|\right)\\ \; & \;-|1\rangle \langle {0|}_{1}⊗|1,1\rangle \langle {0,0|}_{2F}C\left(|r_{1}\rangle \langle r_{0}|\right)+{\left|1\rangle \langle 1\right|}_{1}⊗{\left|1,1\rangle \langle 1,1\right|}_{2F}⊗C(|r_{1}\rangle \langle r_{1}|)), \end{array}$$

upon which Wigner and Bob perform their measurements. The action of the classical channel on the records, again, gives terms of the form

(25) $$\begin{array}{c} \sum_{m}\langle m|r_{i}\rangle \langle r_{j}|m\rangle |m\rangle \langle m|, \end{array}$$

which, if there is no conditioning on the message leads to the effective collapse discussed above regardless of the properties of the channel, i.e., parameters ϕ,θ, since

(26) $$\begin{array}{c} \mathrm{Tr}\left(\sum_{m}\langle m|r_{i}\rangle \langle r_{j}|m\rangle |m\rangle \langle m|\right)=\sum_{m}\langle m|r_{i}\rangle \langle r_{j}|m\rangle =\langle r_{j}|r_{i}\rangle =\delta_{ij}. \end{array}$$

Similar to the probabilities for Wigner’s outcome in the simple Wigner’s friend setup, we can now define the expectation values for the measurements of Bob and Wigner conditioned on the message *n* put out by the classical channel C as follows

(27) $${\langle B⊗W\rangle }^{|n}:=\left\{\begin{array}{cc} \frac{1}{p(n)}\mathrm{Tr}\left(B⊗W⊗|n\rangle \langle n|\cdot \rho \right) & \mathrm{for}\;p(n)>0\\ \;\;\;\;0 & \mathrm{for}\;p(n)=0, \end{array}\right.$$

where the probability for the messages is now given by $p\left(n\right)=\mathrm{Tr}\left(1⊗\left|n\rangle \langle n\right|\cdot \rho_{12FR}^{\prime }\right)=1/2\left(\cos^{2}\left(\theta \right)+\sin^{2}\left(\theta \right)\right)=1/2$. Plugging the state in Equation (24) into this expression, then gives

(28) $$\begin{array}{cc} {\langle B⊗W\rangle }^{|n}= & {\left(\langle 0|B|0\rangle \langle 0,0|W|0,0\rangle |\langle n|r_{0}\rangle \right|}^{2}+\langle 1|B|1\rangle \langle 1,1|W|1,1\rangle {\left|\langle n|r_{1}\rangle \right|}^{2}\\ \; & \;-\langle 1|B|0\rangle \langle 1,1|W|0,0\rangle \langle n|r_{0}\rangle \langle r_{1}|n\rangle -\langle 0|B|1\rangle \langle 0,0|W|1,1\rangle \langle n|r_{1}\rangle \langle r_{0}|n\rangle ). \end{array}$$

For the settings of Bob and Wigner presented in Section 1, we obtain the conditional expectation values

$$\begin{array}{cc} {\langle B_{z}⊗W_{z}\rangle }^{|n} & =\frac{1}{\sqrt{2}}{\left(|\langle n|r_{0}\rangle \right|}^{2}+{\left|\langle n|r_{1}\rangle \right|}^{2})=\frac{1}{\sqrt{2}}={\langle B_{x}⊗W_{z}\rangle }^{|n},\\ {\langle B_{z}⊗W_{x}\rangle }^{|n}= & -\frac{1}{\sqrt{2}}\left(\langle n|r_{0}\rangle \langle r_{1}|n\rangle +\langle n|r_{1}\rangle \langle r_{0}|n\rangle \right)=-{\langle B_{x}⊗W_{x}\rangle }^{|n}. \end{array}$$

**Figure 5:**
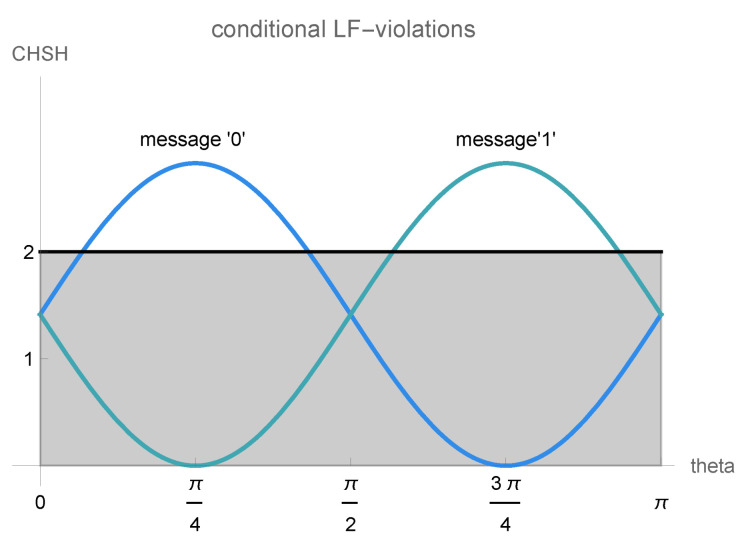
CHSH values for the extended Wigner’s friend setup with communication. The conditional expectation values ${\langle B⊗W\rangle }^{|n}$ for the extended Wigner’s friend experiment in Figure 2 give CHSH values depending on the communication channel parameters ϕ and θ. For ϕ=0 and θ∈[0,π], the expression given by conditioning on message “0” is shown in blue, and the one corresponding to message “1” is depicted in green. There are values of θ where neither of the conditional CHSH values lies above the local friendliness threshold of 2, meaning no violation occurs. Whenever the CHSH-like local friendliness inequality is violated for one of the messages when conditioning on the other message, the local friendliness inequality is satisfied.

Hence, when conditioned on the message *n*, the local friendliness inequality in Equation (22) becomes

(29) $$\begin{array}{cc} {\langle B_{z}⊗W_{z}\rangle }^{|0}+{\langle B_{x}⊗W_{z}\rangle }^{|0}-{\langle B_{z}⊗W_{x}\rangle }^{|0}+{\langle B_{x}⊗W_{x}\rangle }^{|0} & =\sqrt{2}+\sqrt{2}\cdot \cos\left(\phi \right)\sin\left(2\theta \right), \end{array}$$

(30) $$\begin{array}{cc} {\langle B_{z}⊗W_{z}\rangle }^{|1}+{\langle B_{x}⊗W_{z}\rangle }^{|1}-{\langle B_{z}⊗W_{x}\rangle }^{|1}+{\langle B_{x}⊗W_{x}\rangle }^{|1} & =\sqrt{2}-\sqrt{2}\cdot \cos\left(\phi \right)\sin\left(2\theta \right). \end{array}$$

where the term cos(ϕ)sin(2θ) is determined by the properties of the communication channel. If the messages perfectly reveal which result the friend observed, i.e., ϕ=k·π and θ=l·π/2, the channel-dependent term, which corresponds to the expectation values ${\langle B⊗W_{x}\rangle }^{|n}$, vanishes and we obtain the expression in Equation (22) for both messages. If the messages reveal no outcome information about the friend’s measurement, i.e., θ=l·π/4 and ϕ=k·π/2, conditioning on one of the two messages gives the maximal violation of $2\sqrt{2}$. Since the channel-dependent term smoothly varies in the interval [−1,1], one can obtain all values from 0 to $2\sqrt{2}$ for the CHSH-like local friendliness inequality by controlling the channel parameters ϕ and θ. Note that, due to the different signs for the two messages, whenever the conditional expectation values for one message violate local friendliness, the conditional expectation values for the other message do not violate the inequality, compare Figure 5. This is similar to the conditional probabilities p(w|n) for the simple Wigner’s friend experiment discussed in Section 2.2. There, due to the different signs in p(w|0) and p(w|1), these probabilities can exactly reproduce those according to unitary dynamics only for one of the two messages.

## 3. Conclusions

We considered the possibility of communication between Wigner and his friend in both the simple Wigner’s friend scenario and the simplest extended Wigner’s friend setup that allows for the violation of local friendliness inequalities. As we showed explicitly, a classical message revealing which result the friend observed during her measurement effectively collapses the state of her and the system she measured also in the unitary description employed by Wigner. For the simple Wigner’s friend experiment, this means that the probabilities for Wigner’s measurement result according to the unitary description of the setup are the same as those corresponding to collapse dynamics. For the extended Wigner’s friend setup, such records and the corresponding effective collapse prevent the violation of local friendliness inequalities.

We further considered the more general scenario of a (quasi) classical communication channel between Wigner and his friend. In that case, the records the friend produces are the input to the channel while the messages Wigner receives are the output of that channel. Provided that the friend’s records encode which outcome she observed, how much of that outcome information is revealed by the output now depends on the properties of the channel. Controlling the channel parameters then allows for gradual changes between collapse and unitary behavior for Wigner’s friend experiments. In the case of the simple Wigner’s friend experiment, this means that the probabilities based on the unitary description of the setup, associated with Wigner, will gradually approach those assigned by the friend based on the collapse description. For the extended Wigner’s friend scenario, the channel properties determine whether and to what extent local friendliness inequalities can be violated.

Both the recovery of probabilities corresponding to unitary dynamics without records and the maximum violation of local friendliness inequalities not only occur when the messages Wigner receives contain no information about which outcome the friend observed, but also require conditioning on the messages. Simply ignoring the messages put out by the channel always leads to a fully effective collapse. This can be understood as showing that Wigner’s unitary description does not just signify his ignorance about which result his friend observed.

## Data Availability

No new data were created or analyzed. Data sharing is not applicable to this article.
